# Mild Traumatic Brain Injury with Social Defeat Stress Alters Anxiety, Contextual Fear Extinction, and Limbic Monoamines in Adult Rats

**DOI:** 10.3389/fnbeh.2016.00071

**Published:** 2016-04-19

**Authors:** Daniel R. Davies, Dawne Olson, Danielle L. Meyer, Jamie L. Scholl, Michael J. Watt, Pasquale Manzerra, Kenneth J. Renner, Gina L. Forster

**Affiliations:** ^1^Center for Brain and Behavior Research, Division of Basic Biomedical Sciences, Sanford School of Medicine, University of South DakotaVermillion, SD, USA; ^2^Center for Brain and Behavior Research, Department of Biology, University of South DakotaVermillion, SD, USA

**Keywords:** mild traumatic brain injury, social stress, posttraumatic stress disorder, anxiety, fear conditioning, monoamine

## Abstract

Mild traumatic brain injury (mTBI) produces symptoms similar to those typifying posttraumatic stress disorder (PTSD) in humans. We sought to determine whether a rodent model of stress concurrent with mTBI produces characteristics of PTSD such as impaired contextual fear extinction, while also examining concurrent alterations to limbic monoamine activity in brain regions relevant to fear and anxiety states. Male rats were exposed to social stress or control conditions immediately prior to mTBI induction, and 6 days later were tested either for anxiety-like behavior using the elevated plus maze (EPM), or for contextual fear conditioning and extinction. Brains were collected 24 h after EPM testing, and tissue from various limbic regions analyzed for content of monoamines, their precursors and metabolites using HPLC with electrochemical detection. Either social defeat or mTBI alone decreased time spent in open arms of the EPM, indicating greater anxiety-like behavior. However, this effect was enhanced by the combination of treatments. Further, rats exposed to both social defeat and mTBI exhibited greater freezing within extinction sessions compared to all other groups, suggesting impaired contextual fear extinction. Social defeat combined with mTBI also had greater effects on limbic monoamines than either insult alone, particularly with respect to serotonergic effects associated with anxiety and fear learning. The results suggest social stress concurrent with mTBI produces provides a relevant animal model for studying the prevention and treatment of post-concussive psychobiological outcomes.

## Introduction

Mild TBI is one of the most common brain injuries, contributing to 75 percent of total TBI cases (Center for Disease Control and Prevention [CDC], 2003). Clinical evidence suggests mild traumatic brain injury (mTBI) increases the risk for anxiety disorders (Bryant and Harvey, 1999; Moore et al., 2006). This relationship is further exemplified by a recent study of a military population, in which 43.9 percent of those who received a mTBI met the diagnostic criteria for posttraumatic stress disorder (PTSD) compared to just 16.2 percent of those receiving other injuries during deployment (Hoge et al., 2008). In addition, debilitating symptoms of mTBI, including heightened anxiety, can persist for several years post-injury in one third of all patients (Belanger et al., 2007; Kennedy et al., 2007). Given the often persistent nature of mTBI, there is a substantial need for understanding the mechanisms by which mTBI may promote emergence of anxiety disorders, such as PTSD, in order to develop effective preventative treatments.

Comparison of clinical research findings and preclinical animal models of mTBI reveal a substantial overlap between brain regions implicated in PTSD and those most affected by mTBI. For example, smaller hippocampal volume has been associated with PTSD in human studies (Karl et al., 2006; Woon et al., 2010), and animal studies show that mTBI increases cell death and reduces neuronal numbers in the hippocampus (Henninger et al., 2005; Kwon et al., 2011; Dawish et al., 2012; Kovesdi et al., 2012; Meyer et al., 2012). In addition, increased amygdala activation is observed when individuals with PTSD are exposed to reminders of traumatic events (Shin et al., 1997, 2004, 2005; Liberzon et al., 1999; Rauch et al., 2000, 2006; Pissiota et al., 2002; Hendler et al., 2003; Armony et al., 2005; Milad et al., 2006; Morey et al., 2009, 2012), while mTBI causes an increase in the number of neurons in the rat amygdala (Meyer et al., 2012). Furthermore, rats exposed to mTBI also exhibit heightened anxiety-like behavior, as well as enhanced contextual fear conditioning (Meyer et al., 2012) consistent with what has been observed in human studies of PTSD in the laboratory (e.g., Grillon and Morgan, 1999). Therefore, there appears to be some consistency in the alterations to hippocampal and amygdala structure and function that could explain increased anxiety-like signs and fear following mTBI.

However, current preclinical models of mTBI do not show some of the neural and behavioral alterations that often observed in human studies of mTBI or PTSD. For instance, PTSD sufferers exhibit reduced anterior cingulate cortex (ACC) activity when presented with negatively valenced stimuli (Shin et al., 1999; Williams et al., 2006; Kim et al., 2008), and reduced activity in this region is thought to relate to impaired fear extinction (Orr et al., 2000; Milad et al., 2006, 2008; Quirk et al., 2006; Blechert et al., 2007; Jovanovic et al., 2009; Jovanovic and Norrholm, 2011). Furthermore, deficits in medial and dorsolateral prefrontal cortex activity are also believed to contribute to the fear and anxiety symptoms of PTSD (Jovanovic and Norrholm, 2011; Elder et al., 2012; Kovesdi et al., 2012). Clinical evidence suggests that mTBI in humans also results in similar decreases in frontal cortex activity (McAllister et al., 2001; Soeda et al., 2005), while animal models of mTBI have not shown alterations to these frontal regions (e.g., Kwon et al., 2011; Meyer et al., 2012). Moreover, animal studies of mTBI also have not demonstrated altered fear extinction in a manner consistent with exposure to stressors (trauma) and the psychobiological sequelae (Grillon and Morgan, 1999; Meyer et al., 2012; Genovese et al., 2013; Sierra-Mercado et al., 2015). Hence, there is a need to develop a more relevant preclinical model of mTBI with which to examine the neural mechanisms that would promote post-concussive psychological signs.

One important variable that may be missing in the majority of mTBI models is stress/arousal, given that concussive rodent models are induced under anesthesia. Mild TBI is often sustained during times of heightened arousal or stress (combat, domestic violence, accidents, sports), similar to traumatic experiences that can lead to PTSD (Moore et al., 2006). Therefore, we developed a rat model incorporating stress concurrent with the mTBI injury. In this model, we exposed rats to the ethologically relevant stressor of social defeat immediately prior to inducing mTBI to increase stress/arousal at the time of injury. Rodent social defeat is a well-validated paradigm that has been shown to have face, etiological, construct and predictive validity for modeling the neurobiological factors underlying human psychiatric disorders that are promoted by social stress/subordination (Hammels et al., 2015). Rodents exposed to either acute or repeated social defeat from a more aggressive conspecific exhibit many of the same behavioral and physiological responses as elicited by social confrontation in humans, such as anxiety-like behavior, social withdrawal, anhedonia, elevations in sympathetic activity and plasma glucocorticoid release, and changes to monoaminergic activity in stress-responsive limbic regions (Bjorkqvist, 2001; Huhman, 2006; Toth and Neumann, 2013; Hammels et al., 2015). Thus, rodent social defeat has considerable ethological relevance for replicating the biological responses accompanying states of high stress and arousal in humans.

Here we test the hypothesis that combination of social defeat stress and mTBI produces greater increases in anxiety-like behavior, contextual fear conditioning and fear extinction deficits than either treatment alone. Since alterations to monoamines in the limbic system are thought to mediate fear and anxiety states (Millan, 2003; Forster et al., 2012; Hindi Attar et al., 2012; Stockhorst and Antov, 2015), we also examined whether these combined insults affected monoamine levels and activity in limbic regions including the amygdala, hippocampus, and medial prefrontal cortex. Overall, the goal of this study was to determine whether social defeat stress with mTBI could be an effective model for elucidating the neurobiological relationship between mTBI and post-concussive psychobiological signs

## Materials and Methods

### Animals

Adult male Sprague-Dawley rats aged between 8 and 12 weeks (*N* = 106; Animal Resources Center, The University of South Dakota, Vermillion, SD, USA) were used for testing. Animals were housed two per cage after weaning (3 weeks old) and maintained in a reverse light cycle (12 h light/12 h dark), at 22°C, 60% relative humidity with food and water available *ad libitum.* All behavioral testing was performed at least 1 h after the onset of the dark phase (10:00 AM) under red lighting. The experiments were approved by the Institutional Animal Care and Use Committee of South Dakota and the USAMRMC Office of Research Protections Animal Care and Use Review Office, and were conducted in accordance with the National Institute of Health Guide for the Care and Use of Laboratory Animals.

### Social Defeat and Plasma Collection

All rats were acclimated to the social defeat/control testing environment so that any behavioral differences could only be attributable to the social stress pretreatment rather than handling or novel cage exposure. For acclimations, rats were placed individually into an empty cage in the testing room for 40 min for three consecutive days. Twenty-four hours following the last acclimation, rats assigned to the social defeat treatment were transferred into the home cage of a larger resident male, who had been housed in isolation for at least 6 weeks to increase territoriality and aggressiveness (Watt et al., 2009; Novick et al., 2011). Resident males were allowed free contact with the intruder for 10 min and the number of submissions the intruder exhibited in response to resident attacks were recorded (Watt et al., 2009). Following this, a wire mesh barrier was inserted into the cage to separate the two animals from physical contact for 30 min and create a situation of inescapable stress for the intruder (Watt et al., 2009). Defeat intensity (0–5 scale) was calculated for each trial, based on the number of submissions and the overall strength of the defeat. Control animals were placed into novel cages without a resident male for 40 min (Watt et al., 2009). To validate the defeat procedure as reliably eliciting a stress response, a separate group of defeated and control rats (*N* = 10/group) were rapidly decapitated 30 min after the separation period, and blood was collected for plasma measurement of stress-induced corticosterone. This time point matched the time at which mTBI was induced in the following experiments.

### Surgery to Induce mTBI

Within 30 min of the conclusion of social defeat or control procedures, rats underwent sham or mTBI surgery. The mTBI was induced using a mild closed-head weight drop. This procedure produces an injury mimicking a mild concussive injury by altering neuronal number, region volume and number of apoptotic cells in the limbic system, which is accompanied by cognitive deficits, increases anxiety-like behavior and enhanced contextual fear conditioning (Henninger et al., 2005, 2007; Meyer et al., 2012). Weight drop was achieved using a custom-built apparatus (Meyer et al., 2012), consisting of a Plexiglas tube (inner diameter = 11 mm, length = 100 cm) with 5 mm holes drilled every 2 cm to minimize friction and air resistance. Within this tube, a 175 g cylindrical brass weight (10 mm diameter) was held 42 cm above the anesthetized subject’s skull by an electromagnetic switch.

Rats were induced to anesthesia using 4% isoflurane in 3.0 L/min O_2_, with anesthesia maintained at 3% isoflurane delivered through a nose cone. After induction, the rat’s temperature was maintained at 37 ± 0.5°C using a feedback heating pad system (Harvard Apparatus, Holliston, MA, USA). A mid-line scalp incision and fascial clearing was used to expose the skull before placing the anesthetized rat in a prone position under the weight drop device. The animal’s head was secured using two Plexiglas blocks on either side, which also minimized impact-related lateral movement. A cylindrical, polyacetyl transducer rod (diameter = 10 mm, weight = 32.6 g, length = 15.75 cm) was placed in a vertical position in direct contact with the skull, immediately posterior to bregma and centered on the intraparietal suture. Once the anesthetized, animal and transducer rod were properly aligned, the electromagnetic switch was released and the brass weight was dropped directly onto the transducer rod, which transferred the impact to the animal. The transducer rod was immediately grasped after the initial contact to prevent a second impact from recoil.

Rats were returned from the weight drop device to the surgical area to check that no skull fractures resulted from the injury, which would indicate a greater class of injury than mild TBI (i.e., moderate or severe). Once this was assured and visible bleeding had ceased, the wound was closed using wound clips, and the rat removed from anesthesia and administered an analgesic (ketoprofen 5 mg/kg im.). Sham rats underwent the same anesthesia induction and surgical procedures, including being moved to the weight drop device, *sans* weight drop impact. Care was taken to ensure that mTBI and sham groups were under anesthesia for comparable amounts of time to minimize any differences relatable to anesthesia duration (Meyer et al., 2012).

### Measurement of Plasma Corticosterone

Blood was centrifuged (5000 rpm) and the plasma was stored at -80°C until analyzed. Ten microliters of plasma per subject was used in a 100-fold dilution, with plasma corticosterone measured using a corticosterone enzyme linked immunoassay kit (Enzo Life Sciences, Farmingdale, NY, USA) as previously described (Scholl et al., 2009). Samples, assay controls, and standards were run in duplicate. Plasma corticosterone levels were detected by absorbance of samples at 405 nm (wavelength correction set at 595 nm) and compared to known corticosterone standards using an automated plate reader and KinetiCalc Jr. software (Bio-Tek Instruments, Winooski, VT, USA). The detection limit of this assay was 27.0 pg/ml, with sample corticosterone levels expressed as ng/ml of plasma.

### Elevated Plus Maze Testing and Brain Collection

Six days post-surgery, rats (*N* = 10/group) were tested on the elevated plus maze (EPM) as described previously (Meyer et al., 2012). Each arm of the EPM (Noldus Information Technology, Leesburg, VA, USA) was 50 cm long × 12 cm wide, and the maze was elevated 1 m above the ground with a digital camera suspended overhead. Animals were placed in the center of the maze facing toward a closed arm and allowed to explore freely for 5 min. The number of entries into open arms, cumulative time spent in each arm (sec), and total distance moved (cm) was measured using Ethovision XT 5.1 automated tracking software (Noldus Technologies). Twenty-four hours following EPM testing, rats were decapitated, with brains rapidly removed and frozen on dry ice and stored at -80°C.

### Monoamine Analysis

Frozen brains were sliced coronally at -10°C into 300 μm serial sections. Relevant limbic regions were microdissected on a freezing plate (Physitemp Instruments, Inc., Clifton, NJ, USA) using a 20 gage cannula, and included the basolateral/lateral amygdala, central nucleus of the amygdala, medial amygdala, medial prefrontal cortex, dorsal hippocampus, and ventral hippocampus. All samples were expelled into 60 μL sodium acetate buffer (pH: 4.95) containing the internal standard alpha-methyl-dopamine, and stored at -80°C until further analysis.

The monoamines epinephrine, norepinephrine, dopamine, the dopamine metabolite 3,4-dihydroxyphenylacetic acid (DOPAC), the serotonin precursor 5-hydroxytryptamine (5-HTP), serotonin, and the serotonin metabolite 5-hydroxyindoleacetic acid (5-HIAA) were measured as previously described (Scholl et al., 2009; Barr et al., 2013). Briefly, each sample was thawed to lyse cells, had 2 μl ascorbate oxidase (1 mg/ml) added, and then was centrifuged at 15,000 × *g* for 3 min. The supernatant (45 μl) was injected using an Waters 717 Plus Autosampler (Waters Corp., Milford, MA, USA). The monoamines were separated using a Nova-Pak C_18_ 4 μm column (Waters Corp.) and electrochemically detected using an LC-4C detector (BioAnalytical Systems, Inc., West Lafayette, IN, USA) and a glassy carbon electrode set at a potential of +0.6 V vs. an Ag/AgCl reference electrode. The mobile phase contained 14 g citric acid, 8.6 g sodium acetate, 110 mg 1-octane-sulfonic acid, 150 mg EDTA disodium salt, and 100 ml methanol in 1 L deionized water. The pellet was dissolved in 0.4 N NaOH and protein content was measured using the Bradford assay (Bradford, 1976). Monoamine concentrations (pg) were obtained by calculating peak height compared to known standards, and were corrected for recovery using CSW32 v1.4 Chromatography Station for Windows (DataApex, Prague, Czech Republic). The final monoamine value (pg/μg) was obtained by normalizing pg amine to tissue protein content (μg). Measures of dopamine and serotonin activity were approximated as the ratio of DOPAC to dopamine and of 5-HIAA to serotonin, respectively, while capacity for serotonin synthesis was estimated from the ratio of serotonin to 5-HTP.

### Contextual Fear Conditioning and Extinction

Separate groups of rats that did not undergo EPM testing (*N* = 11–12 for each group) were tested for contextual fear conditioning and extinction, beginning 7 days after surgery. Contextual fear conditioning tests took place over 4 days, and followed the procedures of Meyer et al. (2012). On the acquisition day, rats were placed in a foot shock chamber (30 cm × 30 cm; Noldus Information Technology) with an overhead camera within a sound-attenuating chamber (Med-Associates, St. Albans, VT, USA) and were allowed to explore freely for 2 min. After 2 min, a total of 10 electric shocks (0.75 mA, 2 s duration) were delivered at intervals of 74 s through the testing chamber floor (Meyer et al., 2012), with shock delivery controlled by Ethovision 3.1 (Noldus Information Technologies). The rats then remained in the chamber for an additional 2 min with no shocks delivered before being removed. On test days 1–3, rats were placed for 8 min in same chambers as on acquisition day, with no shocks delivered, to determine the extent of contextual fear learning and extinction of contextual fear conditioning. Video footage was later scored for freezing behavior using Ethovision 3.1 by an observer blind to treatment. Freezing behavior was defined as complete immobility except for minor movements required for respiration (Forster et al., 2006).

### Data Analysis

Data from behavioral tests and monoamine concentrations were tested for outliers using the Grubbs’ test prior to analysis. Plasma corticosterone levels were compared between defeated and control rats using one way ANOVA. Behavior on the EPM, monoamines, and unconditioned freezing responses to foot shock during contextual fear acquisition were compared among treatment groups using separate one way ANOVA. Use of one way ANOVA was necessary in order to establish if each treatment alone (mTBI vs. social defeat) produced differential effects, which would not have been possible using a two way ANOVA, where possible interactions between factors preclude such direct pairwise comparisons between stress and mTBI. Significant effects were followed by Student-Newman-Keuls (SNK) *post hoc* tests for multiple comparisons. Furthermore, significant effects of treatment on monoamine levels were followed up with Pearson Product Moment Correlation tests to determine any relationship between the monoamine and time spent in open arms of the EPM. For conditioning tests, within-session and across-session freezing data from the three test days were analyzed among groups for each day using two-way ANOVA with one repeated measure (time), with *post hoc* SNK tests used where applicable. The alpha level was set at 0.05 throughout.

## Results

### Anesthesia and Behavioral Parameters Related to Treatment

There were no significant differences in defeat intensity between social defeat + sham (2.7 ± 0.270) and social defeat + mTBI (2.11 ± 0.269) groups [*F*_(1.43)_ = 2.504, *p* = 0.121]. Furthermore, time under anesthesia during surgery did not significantly differ among all sham (18.023 ± 0.424 min) and all mTBI (17.674 ± 0.369 min) rats [*F*_(1,84)_ = 0.385, *p* = 0.536].

### Plasma Corticosterone Levels Following Social Defeat

**FIGURE 1:**
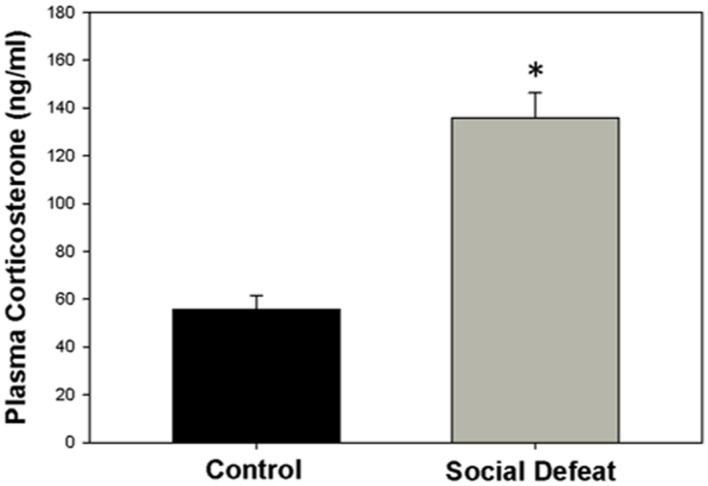
**Plasma corticosterone concentration 30 mins after social defeat or control treatment.** ^∗^ indicates significant difference between groups (*p* < 0.05).

The concentration of plasma corticosterone (ng/mL) was significantly higher in rats exposed to a single episode of social defeat animals than in control animals [*F*_(1,21)_ = 41.547; *p* < 0.001; **Figure 1**].

### Anxiety-like Behavior in Elevated Plus Maze

**FIGURE 2:**
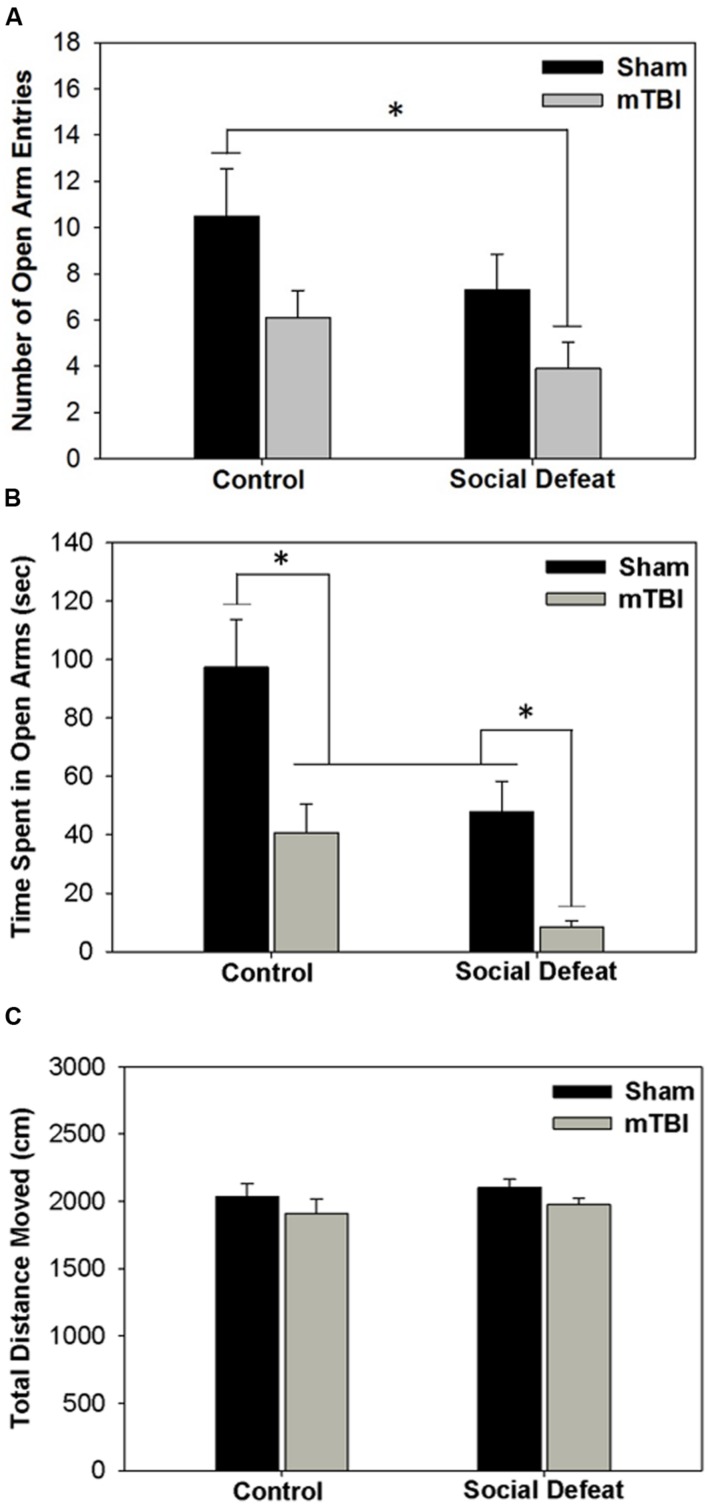
**Behavioral measures from EPM testing 6 days following stress and/or mTBI, including (A) total number of entries into open arms, (B) total time spent in open arms and (C) total distance moved during testing.** ^∗^ Overlying a bar indicates significant difference between individual groups (*p* < 0.05).

Both social defeat and mTBI independently affected the number of entries [**Figure 2A**; *F*_(3,36)_= 3.296, *p* = 0.031] and time spent in open arms of the EPM [**Figure 2B**; *F*_(3,33)_= 10.331, *p* < 0.001]. Rats exposed to social defeat in the absence of mTBI (social defeat + sham) made significantly fewer open arm entries (**Figure 2A**) and spent less time in the open arms (**Figure 2B**), than controls that received sham surgeries (SNK, *p* < 0.05 for both comparisons). Similarly, mTBI treatment in control-handled rats was sufficient to reduce both open arm entry (**Figure 2A**) and duration (**Figure 2B**) compared to sham controls (SNK, *p* < 0.05). Social defeat on its own had equivalent effects to mTBI only, with no difference in either number of entries (**Figure 2A**) or time spent in open arms (**Figure 2B**) between social defeat + sham and control + mTBI groups (SNK, *p* > 0.05). However, the combination of social defeat with mTBI had the greatest effect on anxiety-like behavior, reducing the number of open arm entries not only compared to control + sham treatment but also in comparison to both defeat alone or mTBI alone (**Figure 2A**, SNK, *p* < 0.05 for all comparisons). Likewise, the time spent in open arms was significantly reduced in rats that received both social defeat and mTBI compared with all other groups (**Figure 2B**, SNK, *p* < 0.05 for all comparisons). No difference existed among treatment groups in total distance moved during the EPM test [**Figure 2C**; *F*_(3,36)_ = 1.004, *p* = 0.402].

### Contextual Fear Conditioning and Extinction

**FIGURE 3:**
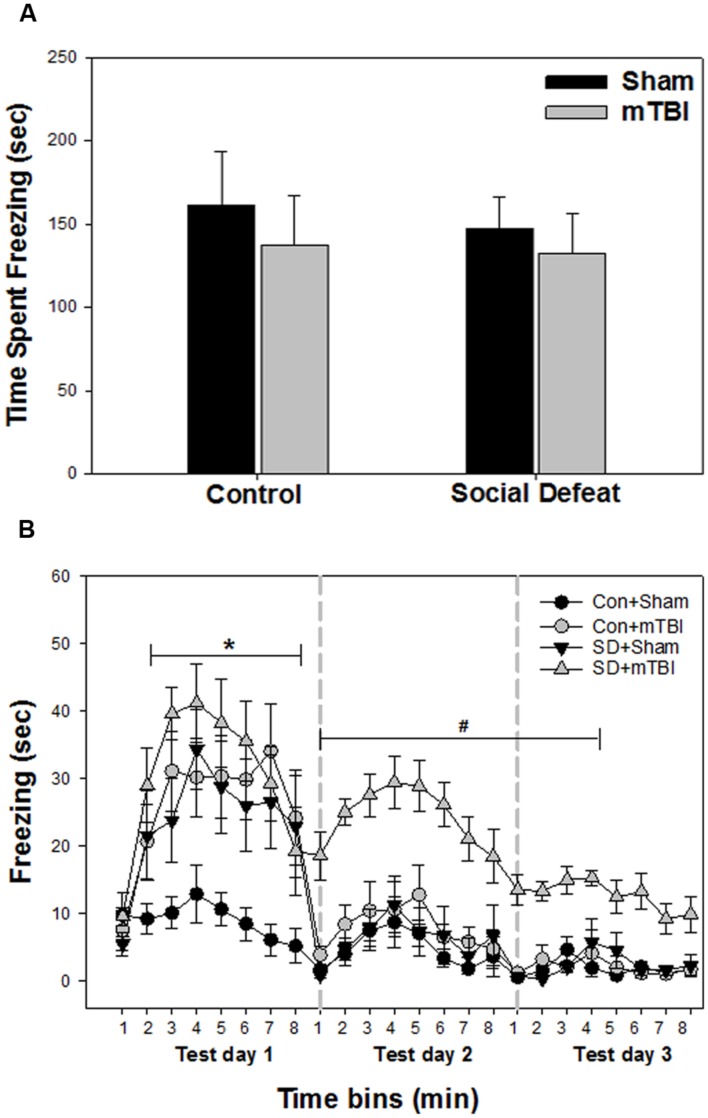
**Freezing behavior from contextual fear conditioning testing beginning 7 days following stress and/or mTBI, including (A) response to footshock during fear conditioning acquisition day and (B) response to context during the three test days in 1 min time bins, each 8 min test separated by 24 h (as indicated by the gray dashed vertical lines).** ^∗^ Indicates significant difference from the control + sham group, while the symbol # indicates significant difference between the social defeat with mTBI vs. all other groups (*p* < 0.05).

Unconditioned freezing duration in response to foot shock was not different between groups [**Figure 3A**; *F*_(3,41)_ = 0.229, *p* = 0.876]. In contrast, two way ANOVA across the three testing sessions revealed a significant main effect of treatment [**Figure 3B**; *F*_(3,40)_ = 13.706, *p* < 0.001], a significant main effect of time [**Figure 3B**; *F*_(23,918)_ = 29.029, *p* < 0.001] and a significant interaction between treatment and time [**Figure 3B**; *F*_(69,918)_ = 2.426, *p* < 0.001] for freezing behavior. All treatment groups (mTBI alone, social defeat alone, and the combined social defeat + mTBI) exhibited more freezing compared to the control + sham group the first test day (SNK, *p* < 0.05 for all eight time bins on test day 1; **Figure 3B**). However, only the combined social defeat + mTBI group showed increased freezing compare to controls during test day 2 (SNK, *p* < 0.05 for all eight time bins on test day 2; **Figure 3B**) and test day 3 (SNK, *p* < 0.05 for the first four time bins on test day 3; **Figure 3B**).

### Monoamine Analysis

**FIGURE 4:**
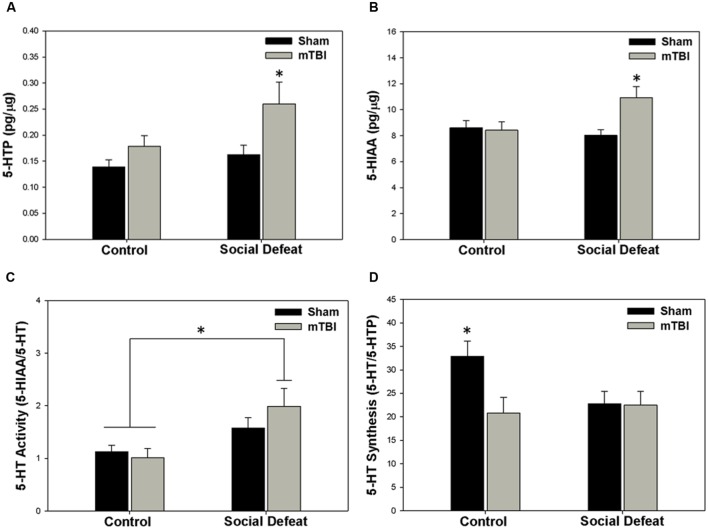
**Serotonin (5-HT) measurements 7 days following stress and/or mTBI, including (A) 5-HTP concentration in the ventral hippocampus, (B) 5-HIAA concentration in the dorsal hippocampus, (C) 5-HT activity in the central amygdala, and (D) 5-HT synthesis in the medial prefrontal cortex.** ^∗^ Indicates significant difference from all other groups, while ^∗^ overlying a bar indicates significant difference between individual groups (*p* < 0.05).

Changes to serotonin function according to treatment were restricted to the hippocampus (dorsal and ventral), the central amygdala, and the medial prefrontal cortex. Alterations in the concentration of 5-HTP were restricted to the ventral hippocampus [**Figure 4A**; *F_(_*_3,43)_ = 4.304, *p* = 0.010], with the social defeat + mTBI group having significantly higher 5-HTP than all other groups (SNK, *p* < 0.027). Similarly, the concentration of 5-HIAA was altered in the dorsal hippocampus [**Figure 4B**; *F*_(3,44)_ = 4.677, *p* = 0.006], with the combined treatment of social defeat and mTBI producing higher 5-HIAA concentrations than all other groups (SNK, *p* < 0.015). Furthermore there was a significant negative correlation between 5-HIAA levels in the dorsal hippocampus and time in open arms of the EPM for social defeat + mTBI group (correlation coefficient = -0.761; *p* = 0.017). In the central amygdala, serotonergic activity (5-HIAA/serotonin) was significantly different among treatment groups [**Figure 4C**; *F*_(3,40)_ = 3.973, *p* = 0.014], with social defeat + mTBI rats showing higher serotonergic activity than both sham + control (SNK, *p* = 0.026) and sham + mTBI (SNK, *p* = 0.019) groups. However, this particular effect appeared to be driven by exposure to social defeat, as changes to central amygdala serotonergic activity were equivalent in all defeated rats with or without mTBI (SNK, *p* = 0.197). Serotonin synthesis capacity (serotonin/5-HTP) was only affected in the medial prefrontal cortex [**Figure 4D**; *F*_(3.40)_ = 3.453, *p* = 0.025], with exposure to either mTBI, social defeat, or a combination of these two factors causing reductions in the serotonin/5-HTP ratio compared to control + sham rats (SNK, *p* = 0.046). There were no differences in either 5-HT concentrations in any brain region assayed (**Table 1**).

**Table 1:** Serotonin in the limbic system 7 days following stress and/or mild TBI.

	*F*-Value	*p*-Value	Mean ± SEM (Pg/μg)			
			Control + Sham	Control + mTBI	SD + Sham	SD + mTBI
**5-HT**						
*mPFC*	0.134	0.939	3.72 ± 0.30	3.56 ± 0.28	3.52 ± 0.33	3.75 ± 0.35
*BLA/LA*	1.902	0.144	14.00 ± 1.25	10.49 ± 0.74	10.32 ± 1.38	12.28 ± 1.52
*CeA*	2.213	0.100	9.33 ± 0.88	10.61 ± 1.23	6.69 ± 0.88	8.28 ± 1.38
*MeA*	0.448	0.720	11.49 ± 1.23	10.34 ± 1.41	9.26 ± 1.38	10.96 ± 1.68
*dHipp*	1.496	0.229	5.88 ± 0.35	5.98 ± 0.35	5.27 ± 0.46	6.50 ± 0.48
*vHipp*	1.675	0.186	6.65 ± 0.35	7.72 ± 0.64	6.17 ± 0.68	6.09 ± 0.60
*dRN*	1.240	0.307	32.95 ± 1.61	29.19 ± 1.64	31.84 ± 1.76	29.38 ± 1.63
**5-HTP**						
*mPFC*	1.698	0.182	0.12 ± 0.02	0.19 ± 0.03	0.17 ± 0.02	0.19 ± 0.03
*BLA/LA*	0.226	0.878	1.15 ± 0.21	1.01 ± 0.13	1.06 ± 0.23	1.23 ± 0.20
*CeA*	1.526	0.223	1.43 ± 0.12	1.48 ± 0.08	2.75 ± 0.78	2.20 ± 0.54
*MeA*	0.498	0.686	1.71 ± 0.06	1.810 ± 0.08	1.81 ± 0.16	1.98 ± 0.29
*dHipp*	1.160	0.337	0.20 ± 0.02	0.18 ± 0.03	0.16 ± 0.03	0.25 ± 0.06
*dRN*	1.259	0.300	5.18 ± 0.21	5.05 ± 0.38	5.55 ± 0.51	4.57 ± 0.28
**5-HIAA**						
*mPFC*	1.085	0.365	4.55 ± 0.26	4.45 ± 0.19	4.64 ± 0.27	5.11 ± 0.37
*BLA/LA*	1.316	0.282	9.63 ± 1.19	8.31 ± 0.72	10.97 ± 1.59	11.86 ± 1.59
*CeA*	0.360	0.782	10.22 ± 0.32	10.13 ± 0.58	10.17 ± 0.69	10.77 ± 0.25
*MeA*	1.290	0.290	10.89 ± 0.57	10.31 ± 0.59	11.50 ± 0.50	11.83 ± 0.69
*vHipp*	1.538	0.219	5.59 ± 0.25	6.59 ± 0.39	6.84 ± 0.31	6.64 ± 0.30
*dRN*	0.700	0.557	22.83 ± 1.33	21.72 ± 0.99	23.62 ± 0.89	21.88 ± 0.99
**5-HT Activity**						
*mPFC*	0.871	0.464	1.26 ± 0.05	1.31 ± 0.08	1.39 ± 0.09	1.44 ± 0.12
*BLA/LA*	1.168	0.333	0.71 ± 0.09	0.99 ± 0.18	1.17 ± 0.26	1.27 ± 0.32
*MeA*	1.080	0.367	1.10 ± 0.16	1.31 ± 0.24	1.81 ± 0.37	1.61 ± 0.38
*dHipp*	0.752	0.527	1.49 ± 0.09	1.43 ± 0.08	1.59 ± 0.08	1.57 ± 0.08
*vHipp*	2.935	**0.045^∗^**	0.92 ± 0.06	0.89 ± 0.08	1.21 ± 0.10	1.12 ± 0.12
*dRN*	1.134	0.346	0.69 ± 0.02	0.754 ± 0.02	0.76 ± 0.03	0.76 ± 0.05
**5-HT Synthesis**						
*BLA/LA*	0.400	0.754	15.50 ± 2.13	12.28 ± 2.38	14.76 ± 2.41	12.69 ± 3.08
*CeA*	1.072	0.372	6.22 ± 1.01	6.76 ± 0.90	3.71 ± 0.96	5.97 ± 2.03
*MeA*	0.145	0.932	6.45 ± 0.66	6.04 ± 1.08	6.15 ± 1.33	5.45 ± 1.16
*dHipp*	0.895	0.452	28.8 ± 4.22	38.45 ± 3.83	28.78 ± 3.29	37.19 ± 8.85
*vHipp*	1.869	0.150	48.39 ± 5.37	43.13 ± 5.16	42.69 ± 6.02	30.50 ± 5.22
*dRN*	0.816	0.492	6.45 ± 0.42	5.95 ± 0.30	6.00 ± 0.31	6.65 ± 0.46

***^∗^**Overall significant difference among groups with ANOVA but no significant pairwise comparisons. 5-HT, Serotonin; 5-HTP, 5-hydroxytryptamine; 5-HIAA, 5-hydroxyindoleacetic acid; mPFC, medial prefrontal cortex; BLA/LA, basolateral/lateral amygdala; CeA, central nucleus of the amygdala; MeA, medial amygdala; dHipp, dorsal hippocampus; vHipp, ventral hippocampus; dRN, dorsal raphe nucleus; mTBI, mild traumatic brain injury; SD, social defeat.*

**FIGURE 5:**
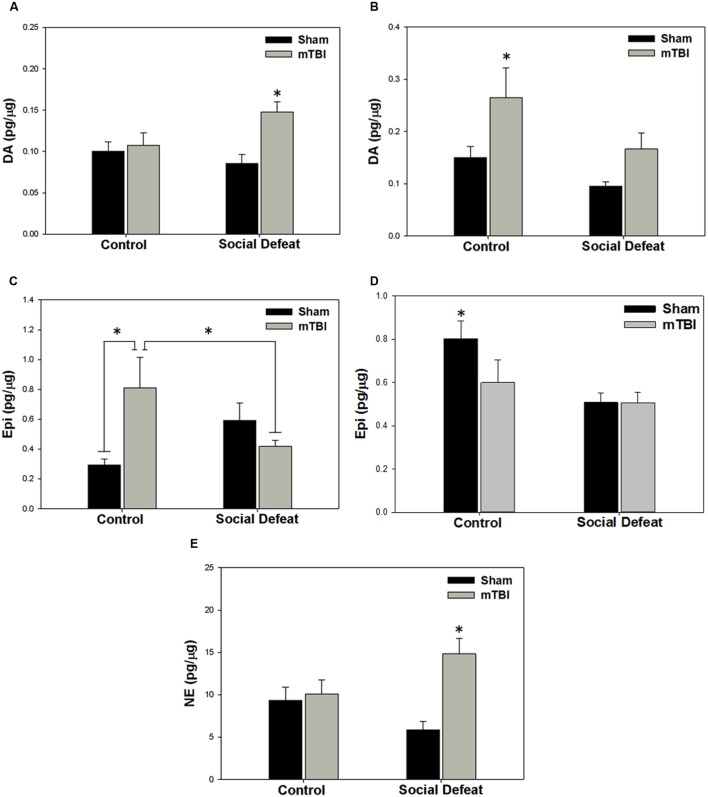
**Catecholamine meatsurements 7 days following stress and/or mTBI, including (A) dopamine (DA) concentrations in the dorsal hippocampus, (B) dopamine concentrations in the ventral hippocampus, (C) epinephrine (Epi) concentrations in the medial amygdala, (D) epinephrine concentrations in the dorsal raphe nucleus, and (E) norepinephrine concentrations in the central amygdala.** ^∗^ indicates significant difference from all other groups, while ^∗^ overlying a bar indicates significant difference between individual groups (*p* < 0.05).

There was a high degree of overlap between regions showing alterations to serotonergic function and those in which catecholamine concentrations were affected. Changes to dopamine concentrations were only seen in the dorsal [**Figure 5A**; *F*_(3,39)_ = 4.758, *p* = 0.006] and ventral hippocampus [**Figure 5B**; *F*_(3,34)_ = 4.404, *p* = 0.01]. Increases in dorsal hippocampus dopamine concentrations were elicited only by the combination of social defeat and mTBI in comparison to all other groups (SNK, *p* = 0.023), while in the ventral hippocampus, only mTBI on its own caused an increase in dopamine relative to the other groups (SNK, *p* = 0.047). Epinephrine in the medial amygdala was differentially altered by treatment [**Figure 5C**; *F*_(3,39)_ = 4.360, *p* = 0.010], with epinephrine concentrations significantly higher in the control + mTBI group when compared to control + sham (*p* = 0.008) and social defeat + mTBI (*p* = 0.041) groups, but not when compared to the social defeat + sham group (*p* = 0.156). The concentration of epinephrine was also significantly different in the dorsal raphe nucleus among treatment groups [**Figure 5D**; *F*_(3,43)_ = 4.354, *p* = 0.009], with all treatments causing reductions in dorsal raphe epinephrine as compared to the control + sham group (SNK, *p* = 0.036). Finally, there was a significant difference among groups in norepinephrine concentration (pg/μg) in the central amygdala [**Figure 5E**; *F*_(3,42)_ = 6.554, *p* = 0.001], with the social defeat + mTBI group exhibiting higher norepinephrine concentrations than all other groups (SNK, *p*-range = 0.001–0.033). There were no differences in either dopamine activity (DOPAC/DA) or DOPAC concentrations in any brain region examined (**Table 2**).

**Table 2:** Catecholamines in the limbic system 7 days following stress and/or mild TBI.

	*F*-Value	*p*-Value	Mean ± SEM (pg/μg)			
			Control + Sham	Control + mTBI	SD + Sham	SD + mTBI
**DA**						
*mPFC*	0.242	0.867	1.33 ± 0.11	1.31 ± 0.12	1.36 ± 0.12	1.44 ± 0.12
*BLA/LA*	0.125	0.945	7.71 ± 0.61	7.87 ± 1.53	8.23 ± 1.46	8.70 ± 1.07
*CeA*	0.442	0.724	14.42 ± 1.97	13.12 ± 2.72	15.43 ± 3.01	11.41 ± 1.69
*MeA*	0.237	0.870	2.17 ± 0.29	1.91 ± 0.27	2.07 ± 0.37	2.28 ± 0.35
*dRN*	1.703	0.180	3.49 ± 0.20	2.97 ± 0.20	3.16 ± 0.15	2.90 ± 0.25
**DOPAC**						
*mPFC*	1.845	0.154	0.49 ± 0.08	0.39 ± 0.03	0.32 ± 0.02	0.35 ± 0.03
*BLA/LA*	1.444	0.243	2.14 ± 0.12	2.40 ± 0.31	2.50 ± 0.26	3.07 ± 0.48
*CeA*	1.452	0.241	2.96 ± 0.34	2.63 ± 0.58	4.22 ± 0.87	4.10 ± 0.72
*MeA*	0.384	0.766	1.53 ± 0.44	1.93 ± 0.16	1.73 ± 0.14	1.86 ± 0.29
*dHipp*	1.228	0.311	0.60 ± 0.05	0.63 ± 0.05	0.71 ± 0.06	0.72 ± 0.07
*vHipp*	1.622	0.198	1.26 ± 0.10	1.21 ± 0.08	1.26 ± 0.10	1.62 ± 0.24
*dRN*	0.257	0.856	0.87 ± 0.06	0.85 ± 0.04	0.89 ± 0.03	0.85 ± 0.03
**DA Activity**						
*mPFC*	0.562	0.643	0.48 ± 0.03	0.50 ± 0.03	0.53 ± 0.05	0.53 ± 0.05
*BLA/LA*	0.936	0.432	0.28 ± 0.01	0.36 ± 0.05	0.33 ± 0.04	0.33 ± 0.04
*CeA*	1.612	0.202	0.23 ± 0.04	0.19 ± 0.02	0.33 ± 0.08	0.33 ± 0.08
*MeA*	1.346	0.295	0.45 ± 0.10	0.92 ± 0.29	0.98 ± 0.24	0.98 ± 0.24
*dHipp*	0.726	0.542	12.42 ± 1.38	10.44 ± 0.77	10.42 ± 1.45	10.42 ± 1.45
*vHipp*	0.952	0.424	2.41 ± 0.49	1.76 ± 0.38	2.61 ± 0.50	2.61 ± 0.50
*dRN*	1.491	0.230	0.25 ± 0.01	0.30 ± 0.02	0.33 ± 0.04	0.33 ± 0.04
**Epi**						
*BLA/LA*	1.632	0.212	0.26 ± 0.06	0.40 ± 0.14	0.17 ± 0.06	0.16 ± 0.04
*CeA*	1.998	0.131	0.28 ± 0.05	0.15 ± 0.03	0.40 ± 0.12	0.61 ± 0.23
*dHipp*	2.001	0.128	0.22 ± 0.01	0.22 ± 0.02	0.20 ± 0.03	0.30 ± 0.05
*vHipp*	0.981	0.411	0.08 ± 0.01	0.08 ± 0.01	0.06 ± 0.01	0.09 ± 0.01
**NE**						
*mPFC*	0.033	0.992	6.03 ± 0.44	5.90 ± 0.39	5.98 ± 0.49	6.09 ± 0.45
*BLA/LA*	0.082	0.970	12.17 ± 1.01	12.19 ± 1.10	11.98 ± 1.16	11.58 ± 0.61
*MeA*	0.847	0.476	11.86 ± 1.44	9.23 ± 1.38	9.39 ± 1.05	10.65 ± 1.44
*dHipp*	2.215	0.100	18.92 ± 0.97	20.79 ± 1.20	17.78 ± 1.61	12.68 ± 4.09
*vHipp*	0.555	0.648	14.18 ± 0.99	15.16 ± 1.18	13.40 ± 0.96	14.37 ± 0.69
*dRN*	1.887	0.146	37.46 ± 2.48	32.53 ± 2.81	31.18 ± 1.93	30.46 ± 1.82

*DA, dopamine; DOPAC, 3,4-dihydroxyphenylacetic acid; Epi, epinephrine; NE, norepinephrine; mPFC, medial prefrontal cortex; BLA/LA, basolateral/lateral amygdala; CeA, central nucleus of the amygdala; MeA, medial amygdala; dHipp, dorsal hippocampus; vHipp, ventral hippocampus; dRN, dorsal raphe nucleus; mTBI, mild traumatic brain injury; SD, social defeat.*

## Discussion

Several behavioral alterations were observed as a consequence of psychosocial stress and/or mTBI in rats. Social defeat or mTBI alone resulted in increased anxiety-like behavior, which was exaggerated when social defeat and mTBI were combined. These effects did not appear to be a result of alterations to locomotion within the EPM, as all treatment groups moved equivalent distance. The findings that either mTBI alone, or a single social defeat, can independently increase measures of anxiety-like behaviors replicates other published work in rodent models (e.g., Berton et al., 1999; Calfa et al., 2006; Baratz et al., 2009; Kwon et al., 2011; Elder et al., 2012; Kinn Rød et al., 2012; Kovesdi et al., 2012; Meyer et al., 2012; Xie et al., 2013; Almeida-Suhett et al., 2014). However, to our knowledge this is the first report of a cumulative effect of combined social stress and mTBI on anxiety-like behaviors, with important implications for how mTBI-related anxiety states could be modeled in rats.

In contrast to the anxiety-like measures, the effects of social defeat and mTBI alone or in combination on contextual fear conditioning were similar. All three treatment groups initially showed greater freezing behavior to the conditioned context than controls with all groups showing equal unconditioned freezing responses to foot shock during the acquisition day. However, rats exposed to the combination of social defeat and mTBI showed poorer extinction within tests sessions 2 and 3 as compared to all other groups, including either social defeat or mTBI alone. While some mTBI models have shown alterations in the acquisition of fear conditioning, all have failed to show extinction deficits in either contextual or cue-based fear conditioning paradigms (e.g., Elder et al., 2012; Meyer et al., 2012; Genovese et al., 2013; Sierra-Mercado et al., 2015). Therefore, the current results suggest that social defeat stress combined with mTBI can elicit post-concussive psychobiological signs that have often not been observed in other mTBI paradigms, but are observed in human laboratory studies of PTSD (Grillon and Morgan, 1999; Steiger et al., 2015). In another model of mTBI with stress, mTBI abolished contextual but not cue fear conditioning as elicited in a rodent PTSD model (Ojo et al., 2014). This study differed from the current study in the mTBI was induced *after* the induction of a PTSD-like model and thus after the acquisition phase of the fear conditioning paradigm, suggesting that the head injury disrupted the consolidation or expression of contextual fear conditioning. The current finding that mTBI current with psychosocial stress potentiates the acquisition of contextual fear conditioning warrants further studies in the timing of the mTBI, along with combining other types of stress with mTBI or with cued fear conditioning. This will determine whether the effects of enhancing acquisition of fear conditioning and impairment of fear extinction are specific to mTBI prior to fear acquisition, specific to concurrent exposure to social defeat, or specific to contextual fear conditioning.

Hypofunction of the prefrontal regions in PTSD is often detected in human neuroimaging studies and is thought to relate to associated impairments in fear extinction (Shin et al., 2001; Milad et al., 2006; Jovanovic and Norrholm, 2011). Using the same mTBI weight drop method employed here, we have shown that neurons are decreased in the dorsal CA1 sub-region of the rat hippocampus and increased in the basolateral/lateral and medial sub-regions of the amygdala following injury (Meyer et al., 2012). These changes are thought to be partly responsible for heightened anxiety and contextual fear conditioning following mTBI (Meyer et al., 2012). This is because contextual fear conditioning is mediated by the dorsal CA1 region of the hippocampus, while the hippocampus does not appear to play a role in acquiring/encoding cue-based fear conditioning (Hunsaker and Kesner, 2008). Similarly, the hippocampus is thought to be important for extinction of contextual fear memory (Milad et al., 2014). However, concussive injury alone does not affect contextual fear extinction in the current and previous studies (Meyer et al., 2012). The expression of contextual fear extinction deficits may also require mTBI-induced impairment of the infralimbic medial prefrontal cortex, as this region in rats, and the equivalent ventromedial prefrontal cortex in humans, appears to work in concert with the hippocampus and the amygdala in contextual fear reinstatement and contextual extinction recall (Milad et al., 2007, 2014; Hitora-Imamura et al., 2015). In line with this, apoptosis or neuronal numbers in any subregion of medial prefrontal cortex of the rat are unaffected by mTBI alone (Meyer et al., 2012). The addition of psychosocial stress at the time of mTBI may increase the susceptibility of the medial prefrontal cortex to enhance apoptotic cell death in this region to contribute to contextual fear extinction deficits. Certainly, the current study shows that corticosterone is elevated by social defeat at the time of mTBI, and previous work has shown that corticosteroid receptors (glucocorticoid and mineralocorticoid) induce apoptotic cell death following brain injury (McCullers et al., 2002). This possibility should be tested by future work.

Social defeat had overlapping effects to those of mTBI alone on altering monoamines in brain regions associated with anxiety-like behaviors, fear conditioning and fear extinction. For example, both treatments independently increased epinephrine in the medial amygdala, and also produced equivalent decreases in dorsal raphe epinephrine and in medial prefrontal cortex serotonin synthesis capacity. While the exact role of epinephrine in the medial amygdala in mediating fearful behavior is unclear, we have shown that mTBI produces increased neuronal numbers and decreased apoptosis in this region (Meyer et al., 2012), along with enhanced contextual fear conditioning equivalent to that seen in defeat only and mTBI alone groups in the current study. In addition, neuronal plasticity in the medial amygdala (as measured by induction of the transcription factor JunB) is required for consolidation of contextual fear memories (Radwanska et al., 2015), and JunB is upregulated by activation of beta-adrenergic receptors in other tissues (Gubits and Yu, 1991; Brand et al., 1993). This raises the possibility that the enhanced conditioned fear responses elicited by either social defeat or mTBI are promoted by epinephrine-mediated increases in medial amygdala plasticity. However, this does not appear to be the case for when these treatments are combined, as rats undergoing both defeat and mTBI showed comparable increases in conditioned fear in the first testing session, but had no change in medial amygdala epinephrine. With regard to changes in the raphe and prefrontal cortex, activation of α1 adrenoreceptors in the dorsal raphe excites serotonergic neurons (Trulson and Crisp, 1984; Clement et al., 1992), suggesting the reduction in prefrontal cortex serotonin synthesis capacity and decreased raphe epinephrine following either social defeat or mTBI may be functionally related. Further, serotonergic lesions of the rat dorsomedial prefrontal cortex enhance contextual fear conditioning (Lehner et al., 2008), so reductions in cortical serotonin synthesis capacity may have contributed to the increased freezing shown by both treatment groups relative to controls (**Figure 3B**). Further testing is required to determine either if cortical serotonin synthesis is indeed reduced following either social defeat or mTBI alone to explain heightened conditioned fear, or whether medial amygdala adrenergic receptor blockade could prevent heightened fear conditioning in these treatment groups.

Exposure to psychosocial stress concurrent with mTBI resulted in a number of alterations to monoamine levels and function that were not apparent with either stressor/insult alone, including increased norepinephrine in the central amygdala and increased dopamine and 5-HIAA concentrations in the dorsal hippocampus. Serotonergic activity as estimated by the 5-HIAA/serotonin ratio was also increased in the central amygdala, although it should be noted that an equivalent effect was induced by social defeat alone. Changes to hippocampal and amygdalar serotonergic activity following combined psychosocial stress and mTBI as compared to stress or mTBI alone may underlie the enhanced anxiety and impaired contextual fear extinction exhibited by these rats. For example, increased 5-HIAA in the hippocampus is associated with increased anxiety (Macbeth et al., 2008), and the current study shows a negative correlation between time spent in open arms and 5-HIAA levels in the dorsal hippocampus supportive of an anxiogenic effect of increased 5-HIAA this region of rats exposed to social defeat and mTBI. Furthermore, the observed heightened serotonergic activity in the amygdala is thought to be anxiogenic (Forster et al., 2012), although its precise role in modulating fear extinction appears dependent upon which serotonin receptor subtypes are activated (Bauer, 2015). Likewise, the ability of concurrent social defeat and mTBI to increase hippocampal serotonin function could underlie deficits in fear extinction (Ohmura et al., 2010). Therefore, the effects of psychosocial stress on outcomes of mTBI may partly be mediated by modifications to limbic monoamines that are not observed in animals subjected to stress or injury alone. These specific changes could offer future targets to ameliorate the negative psychological consequences of mTBI sustained during times of heightened arousal or stress such as occurs in warfare, sporting events, traumatic accidents or domestic violence. One limitation of the current monoamine analysis is the *ex vivo* measure of basal monoamines, and future work should assess *in vivo* monoamine release in the behaving animal, or directly manipulate the monoamines implicated here in specific brain regions to test the role of these in the behavioral outcomes of social stress and mTBI.

## Conclusion

Psychosocial stress combined with mTBI had greater effects on anxiety-like behavior, contextual fear extinction, and limbic monoamine levels or function than either stressor/insult alone. These results suggest that stress or heightened arousal at the time of mTBI augments the psychobiological consequences of concussive injury. Findings from this model suggest future work should focus on the role of monoamines in the hippocampus, prefrontal cortex, and amygdala in moderating the negative psychological consequences of concussive head injury.

## Author Contributions

DD and MW contributed to the design, acquisition, analysis and interpretation of behavioral data. DM and JS contributed to the acquisition and analysis of behavioral and endocrine data. DO and KR contributed to the acquisition, analysis and interpretation of neurochemical data. PM and GF contributed to the design, analysis and interpretation of all aspects of this work. All authors worked on drafting and revising this manuscript, approved the final version for publication, and agreed to be accountable for all aspects of the work.

## Conflict of Interest Statement

The authors declare that the research was conducted in the absence of any commercial or financial relationships that could be construed as a potential conflict of interest.
